# Spontaneous Evisceration Through Umbilical Hernia in Adult Patients: A Scoping Review of Pathophysiology, Clinical Features, Management, and Outcomes

**DOI:** 10.3390/medicina62081590

**Published:** 2026-08-18

**Authors:** Matteo Zanchetta, Natale Calomino, Giuseppe Ietto, Daniele Marrelli, Lorenzo Latham, Silvio Nadalin, Gian Luigi Adani

**Affiliations:** 1 Department of General Surgery (Chirurgia 1), ASST Papa Giovanni XXIII Hospital, 24127 Bergamo, Italy; 2 Kidney Transplant Unit, Department of Medicine Surgery and Neuroscience, University Hospital of Siena, 53100 Siena, Italy; 3 General, Emergency and Transplant Surgery Department, University Hospital of Varese, 21100 Varese, Italy; 4 Surgical Oncology Unit, Department of Medicine Surgery and Neuroscience, University Hospital of Siena, 53100 Siena, Italy; 5 Department of General Surgery, Hospital Moriggia Pelascini, 22015 Gravedona, Italy; 6 Department of General, Visceral and Transplant Surgery, University Hospital of Tübingen, 72016 Tübingen, Germany

**Keywords:** umbilical hernia, evisceration, emergency surgery, abdominal wall, hernia rupture, ascites, cirrhosis, small bowel, bowel resection, coughing

## Abstract

*Background and Objectives*: Few cases of spontaneous evisceration through umbilical hernia (UH) in adult patients have been reported in the literature. Although uncommon, it is a potentially life-threatening surgical emergency. This scoping review aimed to map the published cases and summarize the reported clinical settings, precipitating factors, pathophysiologic mechanisms, management strategies, and outcomes. *Materials and Methods*: A scoping review was conducted in accordance with the PRISMA-ScR guidelines. The search was conducted through PubMed/MEDLINE and Scopus using the strings (“spontaneous evisceration” OR “spontaneous bowel evisceration” OR “umbilical hernia rupture” OR “umbilical hernia evisceration” OR “bowel evisceration umbilical” OR “umbilical evisceration” OR “bowel evisceration” OR “umbilical eventration”). Reports describing spontaneous evisceration of abdominal content through an UH in adult patients were included, whereas pediatric cases, non-umbilical eviscerations, traumatic eviscerations, animal studies, duplicates, and reports without extractable clinical data were excluded. *Results*: From the initial 2623 records identified, after thorough screening and reference tracking of the selected publications, 47 relevant reports for a total of 49 patients were identified. Cirrhosis was reported in 26 patients (53%), umbilical skin changes before the evisceration in 33 (67%), and death in 10 (20%). *Conclusions*: The available evidence suggests that spontaneous evisceration through UH is a surgical emergency that should be viewed not merely as a local mechanical event, but as the result of a multistep process involving chronic pressure overload, tissue fragility, and systemic decompensation, frequently in the setting of cirrhosis with ascites. Urgent repair is usually required, but the role of mesh reinforcement remains unclear and should be individualized according to contamination, defect size, patient condition, and ascites control.

## 1. Introduction

An umbilical hernia (UH) is a ventral midline abdominal wall defect located at or near the umbilicus, from 3 cm above to 3 cm below it, according to the classification of the European Hernia Society [1]. The UH is the second most common hernia in adulthood after inguinal hernia, and in approximately 90% of adult patients it is acquired, rather than congenital [2]. Acute and chronic increases in intra-abdominal pressure, such as strenuous physical exertion, obesity, chronic obstructive pulmonary disease (COPD), or persistent ascites, are critical risk factors for its development. In the general population, the prevalence of UH is approximately 2% [3]. However, in cirrhotic patients with ascites, the prevalence is significantly higher, occurring in around 20% of cases [4], and peaking at approximately 40% in cases of large-volume ascites [5,6]. In instances where the tissues overlying the UH prove incapable of withstanding pressure from within, whether due to chronic conditions and/or a sudden, substantial increase in intra-abdominal pressure, there is the potential for the rupture of the hernia sac and the skin. This may result in the leakage of ascites, a serious complication known as Flood Syndrome, or, in the most severe cases, evisceration of intra-abdominal contents [7,8,9,10]. Ascites develops in more than half of patients with cirrhosis and is indicative of advanced hepatic decompensation, thus rendering it a poor prognostic indicator and thereby accounting for even higher mortality rates in such cases [11]. Although UH is a frequent condition in adults, spontaneous rupture with external evisceration is an uncommon but life-threatening complication. When it occurs, the clinical scenario rapidly shifts from a chronic abdominal wall defect to a potentially lethal surgical emergency. Most available evidence is limited to isolated case reports and small case series, making its management especially challenging. This complication occurs often in patients with liver cirrhosis and tense ascites, in whom chronically increased intra-abdominal pressure progressively enlarges the umbilical defect and thins the overlying skin. Additional factors such as malnutrition, muscle wasting, poor wound healing, ulceration, and continuous ascitic leakage further weaken their abdominal wall and may precede spontaneous rupture. In this fragile population, even minor increases in intra-abdominal pressure, such as coughing, vomiting, or straining, may precipitate evisceration. The management of spontaneous evisceration through a UH is particularly demanding because these patients often present in poor general condition and require urgent treatment in a contaminated or high-risk surgical field. Operative decisions may be difficult, especially regarding bowel viability, the need for resection, the method of fascial closure, and the use or avoidance of mesh in the presence of ascitic leakage and infection risk. Surgery remains the only definitive treatment for UH, although no clear consensus exists regarding the optimal repair technique in this setting. This scoping review aimed to map the published cases of spontaneous evisceration through UH and summarize the reported clinical settings, precipitating factors, pathophysiologic mechanisms, management strategies, and outcomes.

## 2. Materials and Methods

This scoping review was conducted and reported in accordance with the PRISMA Extension for Scoping Reviews (PRISMA-ScR) [12].

The search was conducted in PubMed/MEDLINE using the strings (“spontaneous evisceration” OR “spontaneous bowel evisceration” OR “umbilical hernia rupture” OR “umbilical hernia evisceration” OR “bowel evisceration umbilical” OR “umbilical evisceration” OR “bowel evisceration” OR “umbilical eventration”). Given the rarity of spontaneous evisceration through UH and the predominance of isolated case reports, PubMed/MEDLINE was selected as the primary database because it provides broad coverage of the biomedical and surgical literature, and the electronic search was supplemented by reference tracking of eligible publications to maximize case identification.

To improve the comprehensiveness of the review, the electronic search was subsequently expanded to Scopus using the same search concepts adapted to Scopus syntax. Because this complementary search was performed to identify potentially missed eligible reports, records excluded at this stage were classified globally as not meeting the inclusion criteria and were not further subdivided by exclusion category.

No protocol for this scoping review was prospectively registered. However, eligibility criteria and data-charting items were defined a priori by the authors to ensure methodological rigor. Evisceration was defined as the protrusion of abdominal content from a skin defect overlying UH. We included reports describing spontaneous evisceration of abdominal contents through a UH in adult patients aged 18 years or older. We excluded animal studies, eviscerations through non-umbilical sites, non-relevant articles, pediatric patients, duplicates, and reports without accessible clinical data.

The search was concluded in June 2026 by MZ who independently screened the results and extracted the data into a predefined spreadsheet. Uncertainties were resolved through second opinions by GI, SN, and GLA. PubMed was used as the primary electronic database. Additional sources were identified through reference tracking of eligible publications. Given the descriptive aim of this scoping review and the predominance of case reports and small case series, no formal critical appraisal of individual sources was performed. All eligible reports with extractable clinical data were included to provide a comprehensive map of the available literature.

Data extracted from each included report comprised patient demographics, underlying conditions and comorbidities, precipitating factors, local clinical findings, eviscerated contents, operative management, need for resection, postoperative complications, and reported outcomes. A successful outcome was defined as survival to hospital discharge or reported survival after the index event. For terminological clarity, “rupture” was used to indicate full-thickness disruption of the hernia sac and overlying skin, whereas “evisceration” was defined as external protrusion of intra-abdominal contents, such as omentum or bowel, through the ruptured UH. The term “eventration” was avoided except when referring to historical terminology or search terms, as it was used in older reports to describe presentations consistent with evisceration. Results were synthesized descriptively using counts, percentages, mean values, and ranges. No meta-analysis was performed because of the descriptive aim, the rarity of the condition, and the heterogeneity of the included case reports and case series.

The purpose of the review was to provide a descriptive map of reported clinical patterns, providing surgeons with potential insights regarding the management of these patients, who are often chronically ill and at increased surgical risk due to their comorbidities.

## 3. Results

The search identified 1313 records published in PubMed/MEDLINE. The following exclusions were performed:

- Animal studies (159 records)
- Non-umbilical eviscerations (354 records): transvaginal (190 records); spontaneous transanal (65 records); evisceration resulting from trauma (41 records) among which 16 from abdominal stab wounds (12 from assault, 3 self-inflicted by psychiatric patients, and one resulting from a ritual seppuku), 8 traumatic transanal eviscerations (5 from severe blunt trauma to the abdomen, 2 from uncovered swimming pool drains suction, and one from a vacuum toilet suction), 5 from major burn injuries to the abdomen, 4 from severe blunt trauma to the abdomen, 2 from motor vehicle accidents, 2 traumatic perineal eviscerations resulting from localized severe blunt trauma, 2 from blast injuries to the abdomen, one from a rhino attack, and one from a mountain bike accident; evisceration through wound dehiscence, trocar and drainage sites in the early post-operative period (20 records); parastomal (15 records); evisceration through incisional hernias (14 records); through inguinal hernias (5 records); through crural hernias (one record); trans-stomal (one record); transurethral (one record); and through an enterocutaneous fistula (one record).
- Non-relevant articles (539 records)
- Pediatric patients (221 records)
- Practical exclusions (2): two duplicates.

Fifteen articles that could not be retrieved. Notably, most of the unretrievable reports were old publications (1951, 1953, 1954, 1956, 1960, 1965, 1969, 1972, 1988, and 2004) including one article in Polish (1968), one in Spanish (1980), one in Norwegian (1982), one in Bulgarian (1988), and one in Russian (1988).

Therefore, following the meticulous examination of the initial results and screening according to the predefined criteria, 23 relevant publications were identified, reporting 25 cases. Reference tracking was instrumental in identifying an additional 16 relevant records, for a total of 39 records included.

**Figure 1:**
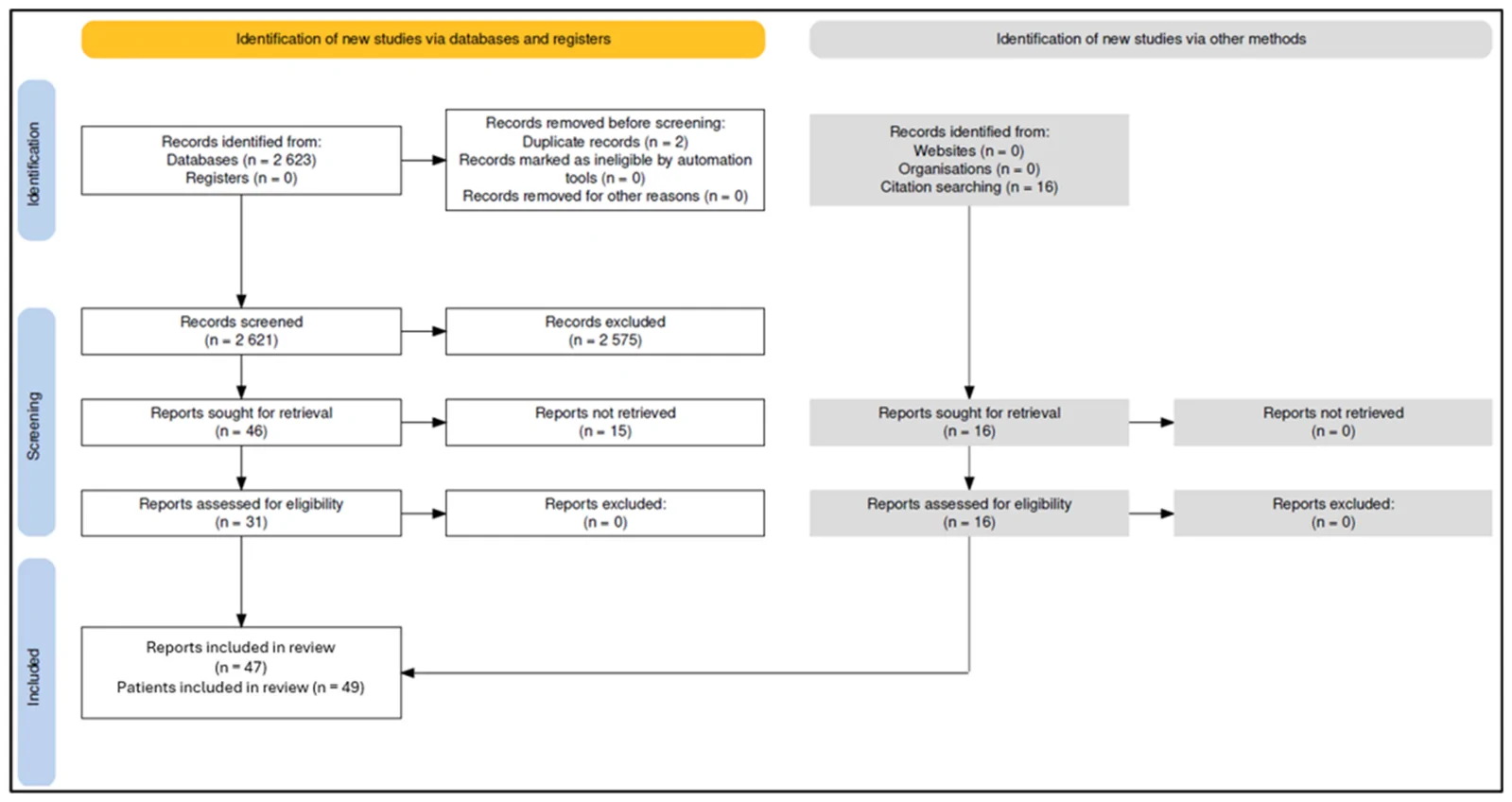
Review flowchart.

The additional Scopus search identified 1310 records, which were screened at the title and abstract level according to the predefined eligibility criteria. After exclusion of records not meeting the inclusion criteria, eight additional eligible single case reports were identified and incorporated into the final dataset, for a total of 49 patients evaluated in the study (Figure 1).

The results represent the largest dataset available on this topic. Main data are summarized in Table 1. The complete patient-level dataset is provided in Supplementary Table S1.

A total of 49 adult patients were included. There were 28 men (57%) and 21 women (43%). Age was reported for 47 patients, with a mean age of 53.1 years and a range of 20–88 years.

**Figure 2:**
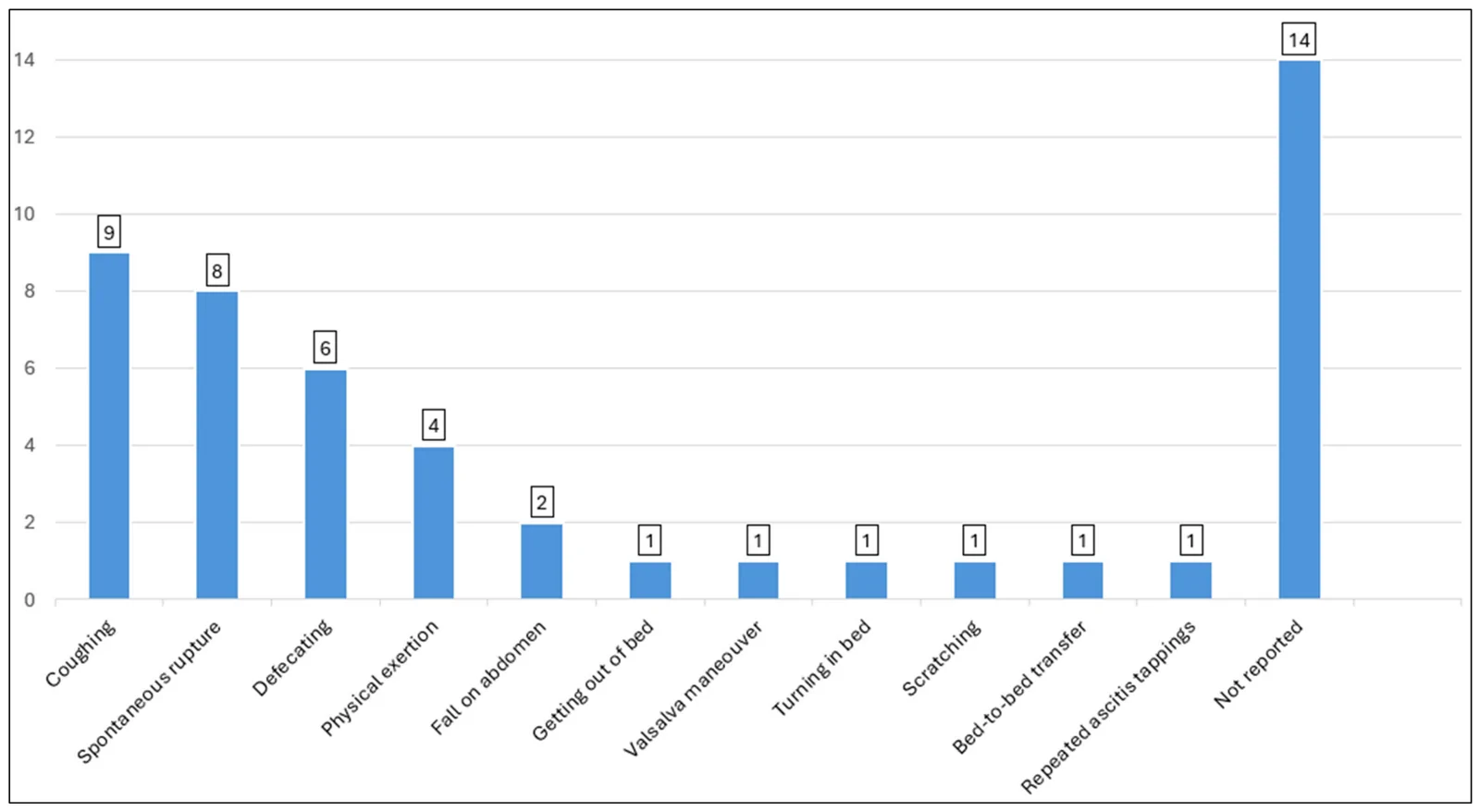
Inciting event: the context within which the evisceration occurred (*n* = 49).

The inciting event was reported in 35 patients (71%) (Figure 2). Coughing was the most frequent precipitating factor (*n* = 9; 26% of reported triggers), followed by spontaneous rupture (*n* = 8; 23%), including two cases occurring during the seventh month of pregnancy. Straining during defecation was reported in six cases (17%), physical exertion in four (11%), and fall or trauma in two (6%). Other isolated triggers included getting out of bed, Valsalva maneuver, turning in bed, scratching, bed-to-bed transfer, and repeated ascitic tapping. In 14 patients (29%), no precipitating event was reported.

Umbilical skin alterations, including erythema, erosion, thinning, ulceration, necrosis, discoloration, or a combination of these findings, were reported in 33 patients (67%). Skin alterations were absent in 13 patients (27%), while this information was not available in three patients (6%).

**Figure 3:**
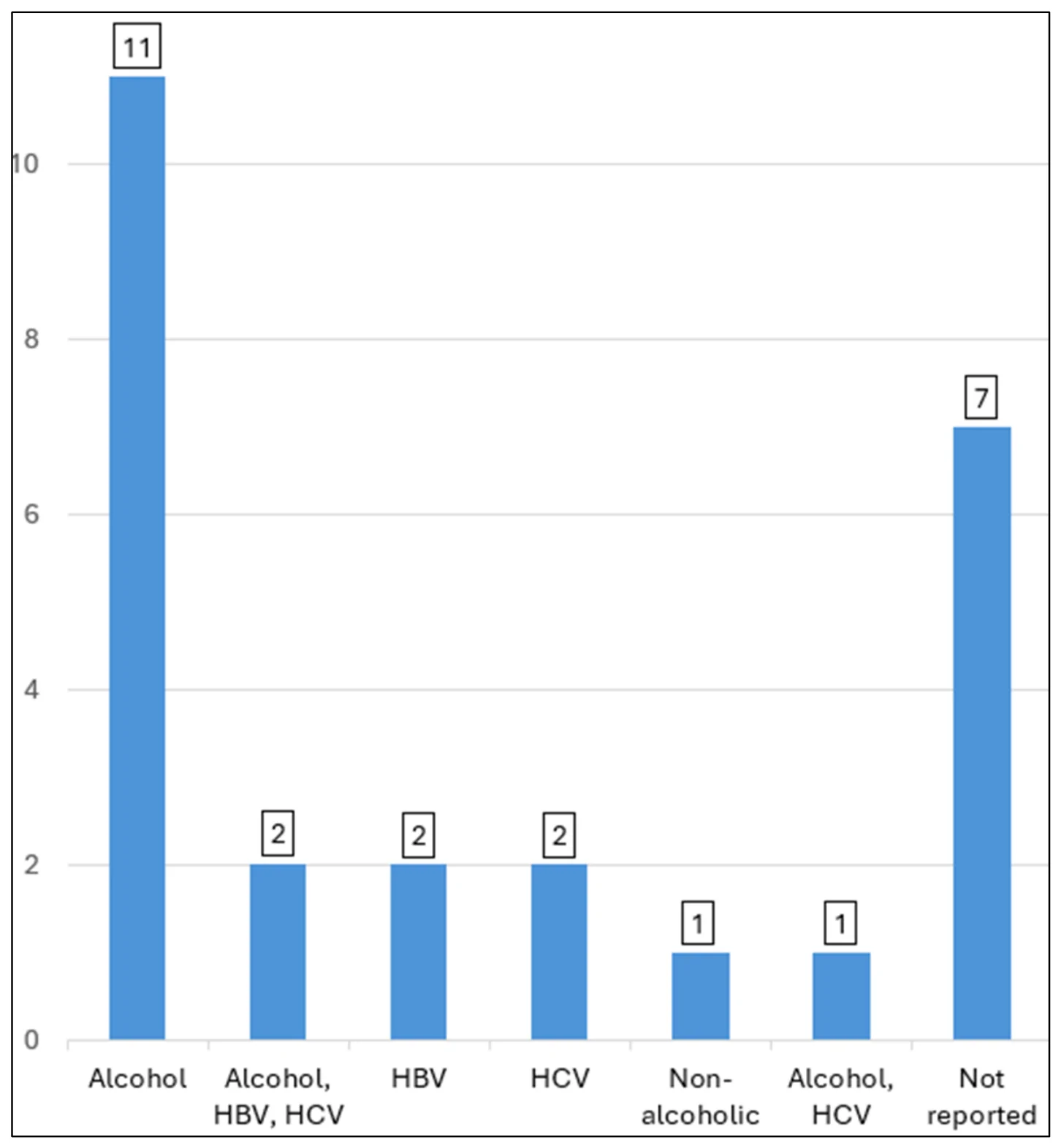
Etiology of cirrhosis (*n* = 26).

Cirrhosis was reported in 26 patients (53%), absent in 20 patients (41%), and not clearly reported in three patients (6%). Child–Pugh class was available in 12 cirrhotic patients: class B in seven and class C in five. Among patients with cirrhosis, the most frequent etiology was alcohol-related liver disease, reported either alone (*n* = 11) or in combination with viral hepatitis (*n* = 3) (Figure 3). Hepatitis B virus was reported in two patients, HCV alone in two patients, and non-alcohol-related cirrhosis in one patient. In seven cirrhotic patients, the etiology was not reported.

Ascites was present in 29 patients (59%). All patients with reported cirrhosis had ascites (26/26; 100%). Ascites was also reported in three patients without definite cirrhosis: one with tuberculous peritonitis, one with an ovarian tumor, and one with chronic liver disease without histologic evidence of cirrhosis.

Leakage before or at the time of rupture was reported in 27 patients (55%). In 24 patients, this represented ascitic leakage in the setting of ascites, while in the remaining three patients, leakage consisted of fecal matter, foul discharge, or local fluid leakage. Among patients with ascites, ascitic leakage was reported in 24 of 29 patients (83%).

Evisceration involved the small bowel alone in 23 patients (47%); the omentum alone in 22 patients (45%); and both small bowel and omentum in three patients (6%), including one cirrhotic and two non-cirrhotic patients. In one cirrhotic patient (2%), the eviscerated content was not specified, although clearly reported as an UH rupture with evisceration. The pattern of evisceration differed according to cirrhosis and ascites status. Among cirrhotic patients, isolated omental evisceration was most common (18/26; 69%), whereas isolated small-bowel evisceration was less frequent (6/26; 23%). By contrast, among non-cirrhotic patients, isolated small-bowel evisceration predominated (14/20; 70%), while isolated omental evisceration occurred in four patients (20%). A similar pattern was observed according to ascites status: isolated omental evisceration occurred in 19 of 29 patients with ascites (66%), whereas isolated small-bowel evisceration occurred in 15 of 20 patients without ascites (75%).

Resection or excision of eviscerated tissue was performed in 27 patients (55%). This consisted mainly of omental resection in 20 patients, while small-bowel resection was performed in seven patients. Small-bowel resection was reported in cases with ischemic, gangrenous, or necrotic bowels.

Direct suture repair without mesh was the most frequently reported approach, performed in 37 patients (76%). Mesh repair was performed in nine patients (18%), including synthetic, biologic, absorbable, or unspecified mesh types. Conservative management was reported in two patients (4%), while the repair strategy was not reported in one patient (2%).

A favorable outcome was documented in 38 patients (78%). Death occurred in 10 patients (20%), while the outcome was not reported in one patient. Mortality was similar among patients with and without cirrhosis, occurring in 5 of 26 cirrhotic patients (19%) and 4 of 20 non-cirrhotic patients (20%). One additional death occurred in a patient for whom cirrhosis status was not clearly reported. Mortality was also similar according to ascites status, occurring in 6 of 29 patients with ascites (21%) and 4 of 20 patients without ascites (20%).

When outcomes were examined according to eviscerated content, death was numerically more frequent after isolated small-bowel evisceration (7/23; 30%) than after isolated omental evisceration (3/22; 14%). No deaths were reported among the three patients with combined small-bowel and omental evisceration. Among patients with reported Child–Pugh class, death occurred in 2 of 5 patients with Child–Pugh C cirrhosis, whereas no deaths were reported among the seven patients with Child–Pugh B cirrhosis. These comparisons were interpreted descriptively only, given the small sample size, incomplete reporting, and case-report-based nature of the available evidence.

## 4. Discussion

To our knowledge, this scoping review represents the most comprehensive synthesis to date of published adult cases of spontaneous evisceration through an umbilical hernia, including 49 patients reported between 1855 and 2026. Although rare, this presentation has persisted across historical and contemporary reports, suggesting that spontaneous evisceration remains a relevant surgical emergency despite advances in perioperative care, hepatology, and abdominal wall surgery. The updated dataset highlights recurrent clinical patterns, including the frequent association with cirrhosis and ascites, the importance of local skin changes and leakage as warning signs, and the need for individualized emergency management.

Considering the available data, cirrhosis was reported in 53% of the patients, and ascites was present in 29 patients (59%). All patients with reported cirrhosis had ascites, supporting the concept that spontaneous evisceration through an UH is often not merely a local abdominal wall event, but part of a broader process of systemic decompensation. In cirrhotic patients, chronic ascites increases intra-abdominal pressure, progressively enlarges the umbilical defect, and places sustained tension on the overlying skin. This mechanical stress is compounded by malnutrition, impaired protein synthesis, sarcopenia, poor wound healing, and general frailty, all of which may contribute to tissue failure and rupture. Among cirrhotic patients, alcohol-related liver disease was the most frequently reported etiology, either alone or in combination with viral hepatitis. This finding should be interpreted cautiously, as it may reflect the high prevalence of alcohol-related advanced liver disease among reported cases rather than an independent association with evisceration. Nevertheless, chronic alcohol exposure may contribute indirectly through cirrhosis, ascites, worsening malnutrition and sarcopenia, inflammation, and impaired tissue repair [13,14,15].

Increased intra-abdominal pressure is central to both the development and progression of UH. In cirrhotic patients, this is mainly driven by ascites, which increases tension on the umbilical ring and contributes to progressive enlargement of the hernia defect. Additional factors, including dilated paraumbilical veins, malnutrition, sarcopenia, and impaired tissue repair, further weaken the abdominal wall. Umbilical hernias are reported in approximately 20% of cirrhotic patients with ascites and may occur in up to 40% of those with large-volume ascites [4,5,6,16,17,18]. Because ascites develops in up to 60% of cirrhosis within ten years from the diagnosis of liver disease, its high prevalence likely explains the strong association between cirrhosis and UH formation [19,20,21].

Evisceration through an UH likely represents the final stage of a multistep pathophysiologic process rather than an isolated local event. Chronic elevation of intra-abdominal pressure, most often related to ascites, progressively enlarges the umbilical defect and places sustained tension on the overlying skin. In cirrhotic patients, this mechanical stress is amplified by malnutrition, sarcopenia, hypoalbuminemia, impaired wound healing, frailty, and portal-hypertension-related changes at the umbilicus, including recanalization and dilatation of paraumbilical veins. Once the overlying tissues become sufficiently fragile, an additional acute increase in intra-abdominal pressure may precipitate rupture and evisceration. In the present review, coughing was the most frequently reported trigger, followed by spontaneous rupture, defecation-related straining, and physical exertion. Two spontaneous ruptures occurred during the seventh month of pregnancy, further supporting the role of increased intra-abdominal pressure in susceptible patients.

Umbilical skin changes, including erythema, erosion, discoloration, thinning, ulceration, necrosis, or a combination of these findings, were reported in 33 patients (67%). These alterations likely reflect progressive weakening of the tissues overlying the hernia, particularly in patients exposed to sustained intra-abdominal pressure from tense ascites or other causes. Ulceration, necrosis, rapid hernia enlargement, and progressive thinning should therefore be regarded as warning signs of impending rupture and should prompt urgent surgical evaluation. Leakage was reported in 27 patients (55%); in 24 cases, this represented ascitic leakage in patients with ascites, while in the remaining three patients, leakage consisted of fecal matter, foul discharge, or local fluid leakage. Among patients with ascites, leakage occurred in 24 of 29 cases (83%), supporting its role as an important red flag for impending hernia rupture or evisceration.

Umbilical hernias may contain preperitoneal fat, omentum, small bowel, or a combination of these structures. If left untreated, they may become incarcerated, strangulated, or, more rarely, rupture with ascitic leakage or external evisceration. Because the neck of the defect is often narrower than the hernia sac, urgent repair is frequently required [22]. In Flood syndrome, surgical management is generally favored when feasible, as conservative treatment has been associated with substantially higher mortality than operative repair [11,23]. In the present review, the eviscerated content differed according to clinical context. Among cirrhotic patients, isolated omental evisceration predominated (69%), whereas isolated small-bowel evisceration was less frequent (23%). Conversely, among non-cirrhotic patients, isolated small-bowel evisceration was more common (70%) than isolated omental evisceration (20%). A similar pattern was observed according to ascites status: omental evisceration predominated in patients with ascites (66%), while small-bowel evisceration predominated in patients without ascites (75%). This distinction may be clinically relevant, as mortality was higher after isolated small bowel evisceration (30%) than after isolated omental evisceration (14%).

Spontaneous rupture with evisceration should be regarded as a surgical emergency. Initial management should include hemodynamic stabilization, sterile coverage of the exposed viscera, broad-spectrum antibiotic therapy, and urgent surgical evaluation. In patients with cirrhosis and ascites, particular attention should be paid to fluid shifts after ascitic loss, intravascular depletion, renal dysfunction, coagulopathy, and infection risk. Intravenous fluids, albumin replacement, correction of coagulation abnormalities, and early ascites control may be required according to the patient’s condition.

Emergency repair in cirrhotic patients is especially challenging because impaired hepatic reserve is often accompanied by thrombocytopenia, hypoalbuminemia, electrolyte disturbances, renal dysfunction, encephalopathy, and tense ascites [24]. The limited time available for optimization increases the risk of bleeding, wound complications, hepatic decompensation, renal deterioration, and postoperative intensive care requirement, with a perioperative mortality increased by five- to tenfold [25]. Therefore, management should be multidisciplinary and individualized, involving surgeons, anesthesiologists, hepatologists, and intensive care specialists whenever possible.

Surgical repair is the only definitive treatment once rupture or evisceration has occurred. In the general population, UH repair is usually associated with low morbidity and recurrence, particularly when mesh reinforcement is used; recurrence rates of 2.7% after mesh repair versus 27% after non-mesh repair have been reported [26,27,28]. In cirrhotic patients, however, the indication and timing of surgery must be balanced against hepatic decompensation, ascites control, and reduced physiological reserve. Recurrence may be particularly high in the setting of uncontrolled or refractory ascites, reaching up to 45% in some reports [4].

In the present review, direct suture repair was the most frequently reported approach, performed in 37 patients (76%), whereas mesh repair was reported in nine patients (18%). This likely reflects the emergency setting, variable contamination risk, bowel or omental viability, tissue quality, and the frequent presence of ascites. Therefore, operative management should be individualized rather than based on a single uniform strategy. Key factors include the patient’s overall condition, comorbidities, size of the fascial defect, viability of the eviscerated contents, degree of contamination, and feasibility of postoperative ascites control. In cirrhotic patients, hepatic reserve, renal function, coagulation status, nutritional status, and the need for multidisciplinary perioperative support should also be carefully considered.

In cases of umbilical skin rupture without evisceration, temporary measures such as glue application may be considered as a short-term bridge to definitive surgery while the patient is optimized [29]. However, once rupture is accompanied by evisceration, management should proceed as an emergency. The immediate priorities are sterile protection of the exposed viscera, assessment of bowel or omental viability, reduction when feasible, and resection of any ischemic, necrotic, or nonviable tissue.

Early elective repair is generally preferable because emergency repair of UH, especially in cirrhotic patients, is associated with worse outcomes, including longer hospital stay, higher readmission rates, and increased short-term mortality [23,30,31,32,33]. In complicated hernias, non-operative management carries particularly high mortality and should generally be reserved for patients who are not candidates for surgery. In unstable patients or contaminated fields, rapid control of the emergency with reduction of viable contents, resection of nonviable tissue when required, and primary suture repair is often the most appropriate approach, particularly for small defects.

Mesh reinforcement may reduce recurrence in selected patients, especially in larger defects, but its use in the emergency setting remains more complex than in elective hernia surgery. Ascites, tissue fragility, contamination, bowel viability, and impaired wound healing may all increase postoperative morbidity [4,34]. Mesh may be placed in an onlay, retromuscular, or intraperitoneal position, depending on local conditions and surgeon preference. In selected patients with larger defects, particularly those exceeding 2 cm, mesh reinforcement may be considered when contamination is limited, tissue quality is acceptable, and postoperative ascites control is achievable. Elective mesh repair has been reported to be feasible and effective in selected cirrhotic patients, with acceptable wound morbidity and lower recurrence [35]. Accordingly, permanent mesh may be considered in selected complicated cases [36], although caution remains warranted because postoperative complications may require mesh removal.

The available case-based evidence remains insufficient to determine the optimal use of mesh, the most appropriate plane of placement, or whether biologic or synthetic mesh provides superior outcomes in this setting. Therefore, decisions regarding mesh use and mesh type should remain individualized, taking into account contamination, bowel viability, defect size, tissue quality, liver function, and the feasibility of postoperative ascites control. Regardless of the repair technique, effective ascites control remains essential to reduce postoperative complications and recurrence after umbilical hernia repair [37].

In the reported cases, repair was performed predominantly by direct suture closure, which was used in 37 patients (76%). Mesh repair was reported in nine patients (18%), whereas two historical cases were managed conservatively and the repair strategy was not reported in one case. One cirrhotic patient underwent bedside omentectomy, ascites drainage, and skin closure because severe coagulopathy precluded emergency laparotomy. Among mesh repairs, the type of prosthesis varied and included synthetic, biologic, absorbable, or unspecified materials. Reported outcomes after mesh use were heterogeneous, ranging from uncomplicated early recovery to wound infection or death from multiorgan failure (MOF). Given the small number of cases, inconsistent reporting, and marked clinical heterogeneity, these data do not allow meaningful comparison between mesh types or placement techniques.

Mortality in this setting should be interpreted in the context of both the acute surgical emergency and the patient’s underlying physiological reserve. Death occurred in 10 patients, and the cause was inconsistently reported. When specified, deaths were attributed to severe systemic complications, including MOF, septic or hemorrhagic shock, acute renal failure after variceal bleeding in decompensated cirrhosis, and postoperative cardiac failure. In some historical cases, the precise cause of death was not clearly described. Although cirrhosis, ascites, hypoalbuminemia, coagulopathy, renal dysfunction, and hepatic decompensation remain important determinants of perioperative risk, several deaths also occurred in non-cirrhotic patients, particularly in association with small-bowel evisceration, ischemia, or severe emergency presentation. These observations suggest that outcome is unlikely to depend on a single factor, but rather on the interaction between systemic vulnerability, viability of the eviscerated contents, contamination, delay to treatment, and the feasibility of effective perioperative optimization. Therefore, spontaneous evisceration through a UH should be regarded as a high-risk emergency requiring rapid source control, careful assessment of bowel viability, and individualized multidisciplinary management, especially in patients with advanced liver disease or poor physiological reserve.

This scoping review has several limitations. First, the available evidence consists predominantly of isolated case reports and small case series, which limit the strength, generalizability, and comparability of the findings. Moreover, several included reports were historical case descriptions, in which diagnostic work-up, perioperative management, terminology, and outcome reporting differed substantially from contemporary standards. Therefore, the available evidence should be considered low-level. The findings of this review should consequently be interpreted as descriptive patterns rather than definitive evidence. Second, reporting across the included sources was heterogeneous, and several clinically relevant variables were inconsistently described. Third, the screening process was primarily performed by one reviewer, with uncertainties resolved through consultation, rather than by fully independent duplicate screening from the outset. Fourth, because of the rarity of spontaneous evisceration through an UH and the descriptive nature of the available literature, no formal meta-analysis or robust comparative assessment of treatment strategies was feasible. Nevertheless, by methodically mapping the reported cases and recurrent clinical patterns, this review may provide practical guidance for clinicians facing this rare but life-threatening surgical emergency. Larger multicenter registries or collaborative case collections may help refine management recommendations, although the rarity of this event makes prospective studies difficult.

## 5. Conclusions

Spontaneous evisceration through an UH is a rare but life-threatening surgical emergency, frequently occurring in patients with cirrhosis, ascites, or other causes of chronically increased intra-abdominal pressure. Umbilical skin changes, including thinning, ulceration, discoloration, or necrosis, and ascitic leakage should be regarded as warning signs of impending rupture and should prompt urgent surgical evaluation. Once evisceration occurs, management should be individualized according to the patient’s clinical condition, viability of the eviscerated contents, degree of contamination, fascial defect size, tissue quality, liver function, and feasibility of postoperative ascites control. Before rupture occurs, elective repair after optimization and ascites control is preferable in selected cirrhotic patients, because emergency repair is associated with greater morbidity. In the emergency setting, primary suture repair remains the most reported approach, whereas mesh repair should be considered only in carefully selected cases. Overall, spontaneous evisceration should be viewed as the final clinical expression of progressive tissue failure under sustained intra-abdominal pressure, and effective ascites control remains essential to improve outcomes and reduce recurrence.

## Figures and Tables

**Table 1:** Summary of clinical characteristics, management, and outcomes.

Variable	Value
Total patients	49
Male sex	28 (57%)
Age (*n* = 47), mean (range)	53.1 years (20–88)
Inciting event reported:	35/49
- Coughing	- 9/35 (26%)
- Spontaneous	- 8/35 (23%)
- Defecating	- 6/35 (17%)
Umbilical skin alterations	33 (67%)
Cirrhosis	26 (53%)
Ascites	29 (59%)
Leakage before rupture	27 (55%)
Viscus eviscerated:	
- Omentum (O)	22 (45%)
- Small bowel (SB)	23 (47%)
- O and SB	3 (6%)
- Not reported	1 (2%)
Resection/excision	27 (55%)
Management:	
- Direct repair	37 (76%)
- Mesh repair	9 (18%)
- Conservative	2 (4%)
- Not reported	1 (2%)
Outcome:	
- Favorable	38/49 (78%)
- Death	10/49 (20%)
- Not reported	1/49 (2%)

## Data Availability

The data collected for this study are presented and included in this article. Further inquiries can be directed to the corresponding author.

## Supplementary Materials

The following supporting information can be downloaded at: https://www.mdpi.com/article/10.3390/medicina62081590/s1. The complete patient-level dataset is provided in Supplementary Table S1 on the online version of the manuscript.
