# Supplementary material for: Spontaneous Evisceration Through Umbilical Hernia in Adult Patients: A Scoping Review of Pathophysiology, Clinical Features, Management, and Outcomes
Source: Medicina (Kaunas). 2026 Aug 18;62(8):1590. doi: 10.3390/medicina62081590 (PMC13515857; doi:10.3390/medicina62081590)
Supplement: Supplementary file 1 [file medicina-62-01590-s001.zip › medicina-4354810-supplementary.pdf]

SUPPLEMENTARY TABLE S1 : PATIENT-LEVEL DATASET WITH RESULTS OF THE REVIEW

Legend:

- A : alcohol
- Co : conservative management
- CP : Child Pugh
- D : direct repair of the hernial defect with sutures, without mesh
- HBV : Hepatitis B Virus
- HCV : Hepatitis C Virus
- M : repair of the hernial defect using a mesh (either biologic or synthetic, when reported)
- O : omentum
- S : small bowel
- Data not reported are represented as "-"

Cirrhosis: when available, the CP score is reported after "yes"; the etiology of cirrhosis is reported in brackets, when available.

Inciting event: when reported, the context within which the evisceration occurred.

Leakage: unless otherwise specified, reported leak of ascites before or contextual to the rupture and evisceration.

Skin alterations: when available data, the reported presence or absence of skin alterations over the umbilical hernia.

Author	Year	Sex	Age	Inciting event	Skin alterations	Leakage	Ascites	Cirrhosis +/- CP (cause)	Eviscerated viscus	Management	Resection	Outcome
Porter [1]	1855	Female	65	Fall	Yes	Fecal matter	No	-	S	Co	No	Discharged
Bontecou [2]	1858	Female	-	Physical exertion	Yes	No	No	No	S+O	Direct	No	Discharged
Benson [3]	1865	Male	50	Physical exertion	Yes	No	No	-	S	Co	No	Discharged
Unknown [4]	1882	Female	50	Fall	Yes	No	No	-	S	D	No	Deceased
Lawson [5]	1890	Female	-	Defecating	Yes	No	No	No	S	Direct	No	Deceased
Johnson [6]	1901	Female	30	Spontaneous	No	Yes	Yes	Yes	O	D	Yes	Discharged
Travers [7]	1902	Female	60	Coughing	No	No	No	No	S	Direct	Yes	Deceased
McGrath [8]	1918	Female	78	Defecation	No	No	No	No	S + O	D	Yes	Discharged
Glanville [9]	1955	Female	30	Spontaneous, 7th month pregnant	No	No	No	No	O	D	Yes	Discharged
Hardinger [10]	1959	Male	71	Getting out of bed	Yes	No	No	No	S	D	No	Discharged
Francis [11]	1960	Male	68	Spontaneous	Yes	No	Yes	No	O	D	Yes	Discharged

Mays 12]	1969	Female	20	Spontaneous	Yes	No	No	No	S	D	No	Discharged
Bhandari [13]	1972	Male	50	Coughing	No	Yes	Tuberculous peritonitis	No	S	D	No	Deceased
Koishi [14]	2003	Female	81	Defecation	No	No	Ovarian tumor	No	S	D	Yes	Discharged
Perriello [15]	2004	Male	72	Valsalva maneuver	No	No	No	No	S	Synthetic M	No	Discharged
Ginsburg [16]	2006	Male	45	Turning in bed	Yes	No	Yes	Yes (A, HBV, HCV)	O	D	Yes	Discharged
Shrestha [17]	2007	Male	51	Spontaneous	Yes	Yes	Yes	Yes	O	M	Yes	Discharged
Choo [18]	2008	Male	41	Coughing	No	Yes	Yes	Yes (A)	S + O	D	No	Discharged
Miryala [19]	2009	Male	68	Coughing	Yes	Yes	Yes	Yes (A)	O	D	Yes	-
Kinuda [20]	2010	Male	54	-	-	Yes	Yes	Yes (A)	O	D	Yes	Discharged
Ahmed [21]	2011	Female	28	Spontaneous, 7th month pregnant	Yes	No	No	No	S	D	Yes	Discharged
Good [22]	2011	Male	81	Defecation	Yes	Yes	Yes	Yes (A)	O	D	Yes	Discharged
Buffone [23]	2012	Male	61	Spontaneous	Yes	Yes	Yes	Yes (A)	O	D	Yes	Deceased
Descloux [24]	2012	Female	45	Coughing	No	Yes	Yes	Yes (C)	O	D	No	Discharged
Kato [25]	2013	Female	88	Scratching	Yes	No	No	No	S	D	Yes	Discharged
Laucho [26]	2013	Male	58	-	Yes	Yes	Yes	Yes B (A)	O	D	No	Discharged
Ogu [27]	2013	Male	50	Coughing	Yes	Yes	Yes	Yes (A, HCV)	S	Biologic M	Yes	Discharged
Ferreira [28]	2014	Female	54	-	Yes	Foul discharge	No	No	S	Synthetic M	Yes	Discharged
Arora 1 [29]	2018	Female	42	-	Yes	Yes	Yes	Yes C (HCV)	O	D	Yes	Deceased
Arora 2 [29]	2018	Male	40	-	Yes	Yes	Yes	Yes C (A)	O	D	No	Deceased
Colbran [30]	2019	Male	59	Coughing	Yes	Yes	Yes	Yes B (A, HBV, HCV)	O	D	Yes	Discharged
Lim [31]	2020	Male	65	Bed to bed transfer	Yes	Yes	Yes	Yes B (HBV)	S	Biologic M	No	Discharged
Jeuken [32]	2020	Female	57	-	Yes	Yes	Yes	Yes C	O	D	No	Discharged
Balu [33]	2021	Male	42	Physical exertion	Yes	Yes	No	No	O	D	Yes	Discharged
Bolivar-Rodriguez [34]	2021	Male	46	Physical exertion	Yes	No	No	No	S	D	No	Discharged
Albeladi [35]	2021	Female	23	Multiple ascites	Yes	Yes	Yes	Yes B (HBV)	O	Synthetic M	Yes	Discharged

				tapping								
Limbu [36]	2021	Female	43	Coughing	Yes	No	No	No	S	D	No	Discharged
Patel [37]	2021	Male	70	-	No	Yes	Yes	Yes (non-A)	O	D	Yes	Discharged
Iizuka [38]	2022	Female	42	Defecation	No	Yes	Yes	Yes C (A)	S	D	No	Discharged
Nugroho [39]	2022	Male	49	-	Yes	Yes	Yes	Yes	S	M	No	Discharged
Tuñón-Féquant [40]	2023	Male	58	-	-	No	Yes	Yes (A)	-	M	No	Discharged
Grappolini [41]	2024	Male	53	Coughing	Yes	No	No	No	S	D	Yes	Discharged
Jayaprakash [42]	2024	Male	55	-	Yes	Yes	Yes	Yes B (A)	O	D	No	Discharged
Aldemyati [43]	2025	Male	50	-	Yes	No	No	No	O	D	Yes	Discharged
Barden [44]	2025	Female	61	Spontaneous	-	Yes	Yes	Yes (A)	S	Absorbable M	No	Deceased
Bruton [45]	2025	Male	47	Defecation	Yes	No	No	No	S	D	Yes	Deceased
Singh 1 [46]	2025	Female	58	-	No	Yes	Yes	Yes B (HCV)	O	D	Yes	Discharged
Singh 2 [46]	2025	Male	52	-	No	Yes	Yes	Yes B	O	D	Yes	Discharged
Dubovský [47]	2026	Male	36	-	Yes	No	Yes	Yes	S	-	Yes	Deceased

REFERENCES

1. Porter, H. Umbilical hernia, with rupture of the intestine and subsequent closure of the opening. Assoc. Med. J. 1855, 3, 1108–1109. https://doi.org/10.1136/bmj.s3-3.154.1108
2. Bontecou RB. Rupture of an umbilical hernia, and escape of a large mass of intestines—peritonitis—opium—recovery. Am Med Mon. 1858;10:341–344
3. Benson. Umbilical hernia; sloughing of four inches of the small intestines; complete recovery. Dubl. Med. Press 1865 Dec;22(12):570-571
4. Anonymous. Rupture of an umbilical hernia, with prolapsus of intestines. Med. Chir. Centralbl. 1882.
5. Lawson G. Middlesex Hospital: Rupture of the coverings and protrusion of bowel from an old umbilical hernia; operation; death. Lancet. 1890;136(3498):562.
6. Johnson, J.T. Ruptured umbilical hernia. Trans. South. Surg. Assoc. 1901, 14, 257–268.
7. Travers FT. Rupture of the coverings of an umbilical hernia with external protrusion of the intestine. Lancet. 1902;159(4098):739.
8. McGrath WJ. Unusual case of umbilical hernia: rupture of skin. JAMA.1918;71(20):1650. doi: 10.1001/jama.1918.26020460001009
9. De Glanville H. Ruptured umbilical hernia. Lancet. 1955 Dec 24;269(6904):1321. doi: 10.1016/s0140-6736(55)93115-9. PMID: 13272384.
10. Hardinger JD, Noskin EA. Spontaneous eventration through the umbilicus; report of a case. N Engl J Med. 1959 Jul 16;261(3):141-2. doi: 10.1056/NEJM195907162610308. PMID: 13666995.
11. Francis WW. Spontaneous rupture of herniae. Rhode Island Medical Journal. 1960;43:39–40.
12. Mays ET. Eventration through umbilical hernia during pregnancy. J Ky Med Assoc. 1969 Feb;67(2):110-2 passim. PMID: 5763698.
13. Bhandari NS, Shastri KD, Sharma AK. Spontaneous rupture of umbilical hernia with evisceration of small intestine. J Indian Med Assoc. 1972 Sep 16;59(6):243-4. PMID: 4658698.
14. Koishi, K.; Kusuhara, K.; Yagyu, R.; Fujiwara, Y.; Nakao, K.; Nakagawa, K.; Yamamura, T. An adult case of umbilical hernia incarceration combined with perforation of hernia sac. J. Jpn. Coll. Surg. 2003, 28, 903–906.
15. Perriello J, Pérez-Catón R, Mendiburu A. Hernia umbilical eviscerada. Revista del Hospital Privado de Comunidad 2004; 7(2):27-28.

16. Ginsburg BY, Sharma AN. Spontaneous rupture of an umbilical hernia with evisceration. J Emerg Med. 2006 Feb;30(2):155-7. doi: 10.1016/j.jemermed.2005.05.017. PMID: 16567249.
17. Shrestha DB, Tsang A, Courtney ED, Pittam M, Ravichandran D. Spontaneous rupture of an umbilical hernia. Br J Hosp Med (Lond). 2007 Oct;68(10):561. doi: 10.12968/hmed.2007.68.10.27332. PMID: 17974309.
18. Choo EK, McElroy S. Spontaneous bowel evisceration in a patient with alcoholic cirrhosis and an umbilical hernia. J Emerg Med. 2008 Jan;34(1):41-3. doi: 10.1016/j.jemermed.2007.03.035. Epub 2007 Aug 29. PMID: 17976794.
19. Miryala R, Neilan R. Images in clinical medicine. Perforated umbilical hernia. N Engl J Med. 2009 Jun 18;360(25):e32. doi: 10.1056/NEJMicm0801211. PMID: 19535799
20. Kinuda, S.; Koshiishi, N.; Matsumura, Y.; Hirai, Y.; Watanabe, H.; Kijima, Y. A case of ruptured umbilical hernia present concomitantly with prolapse of the omentum due to intractable ascites in an adult. J. Jpn. Surg. Assoc. 2010, 71, 221–224. https://doi.org/10.3919/jjsa.71.221
21. Ahmed A, Stephen G, Ukwenya Y. Spontaneous rupture of umbilical hernia in pregnancy: a case report. Oman Med J. 2011 Jul;26(4):285-7. doi: 10.5001/omj.2011.70. PMID: 22043438; PMCID: PMC3191721
22. Good DW, Royds JE, Smith MJ, Neary PC, Eguare E. Umbilical hernia rupture with evisceration of omentum from massive ascites: a case report. J Med Case Rep. 2011 May 3;5:170. doi: 10.1186/1752-1947-5-170. PMID: 21539740; PMCID: PMC3095553
23. Buffone A, Costanzo M, Basile G, Terranova L, Papa V, Catania A, Cannizzaro MA. Spontaneous rupture of an umbilical hernia in a cirrhotic patient with ascites A case report and review of the literature. Ann Ital Chir. 2012 Oct 29;2012:S2239253X1202021X. PMID: 23160169.
24. Descloux, A.; Kocher, T. Wie lautet Ihre Diagnose? Praxis 2012, 101, 1471–1472. https://doi.org/10.1024/1661-8157/a001107
25. Kato, T.; Terasaki, M.; Okamoto, Y.; et al. An adult umbilical hernia with spontaneous evisceration of the small intestine. J. Jpn. Soc. Abdom. Emerg. Med. 2013, 33, 1067–1070.
26. Laucho, T.; Velásquez, J.; Díaz, H. Hernia umbilical eviscerada en pacientes cirróticos: Reporte de un caso. Gen 2013, 67, 156–159.
27. Ogu US, Valko J, Wilhelm J, Dy V. Spontaneous evisceration of bowel through an umbilical hernia in a patient with refractory ascites. J Surg Case Rep. 2013 Oct 4;2013(10):rjt073. doi: 10.1093/jscr/rjt073. PMID: 24964319; PMCID: PMC3852797.
28. Ferreira, G.S.A.; Nakadomari, T.; Tabushi, F.L.; Tabushi, F.I.; Marques, D.M.R.; Polonio, B. Hérnia umbilical gigante eviscerada: Relato de caso. Rev. Méd. UFPR 2014, 1, 166–168. https://doi.org/10.5380/rmu.v1i4.40696
29. Arora E, Gandhi S, Bhandarwar A, Quraishi AHM, Wagh A, Tandur A, Wakle D. Umbilical Hernia with Evisceration. Two Cases and a Review of the Literature. J Emerg Med. 2018 Aug;55(2):e27-e31. doi: 10.1016/j.jemermed.2018.04.036. Epub 2018 May 21. PMID: 29793813.
30. Colbran, R.; Smith, A.; Melloy, A.; Iyer, R. Spontaneous umbilical hernia rupture with omental evisceration and Flood syndrome. Int. Surg. J. 2019, 6, 3830–3833. https://doi.org/10.18203/2349-2902.isj20194450
31. Lim, E.W.; Ngaserin, S.; Foo, F.J. Inside-out: Spontaneous bowel evisceration, a rare complication of the ventral hernia. Int. J. Abdom. Wall Hernia Surg. 2020, 3, 155–157. https://doi.org/10.4103/ijawhs.ijawhs_25_20
32. Jeuken RM, Broos PPHL, Belgers EHJ. Een vrouw met vetweefsel uit haar navel [Protruding omentum with umbilical fluid leakage in a patient with liver cirrhosis]. Ned Tijdschr Geneeskd. 2020 Apr 30;164:D4716. Dutch. PMID: 32395961.
33. Balu P, et al. Spontaneous evisceration of adult umbilical hernia: case report. Surg Chron. 2021;26(3):343–344.
34. Bolívar-Rodríguez, M.A.; Magaña-Zavala, P.A.; Pamanes-Lozano, A.; et al. Evisceración por rotura espontánea de hernia umbilical en adulto. Cir. Esp. 2021, 99, 687. https://doi.org/10.1016/j.ciresp.2020.10.015
35. Albeladi, A.M.; Odeh, A.M.; AlAli, A.H.; Alkhars, A.M.; Buhlaigah, A.M.; Alghadeer, H.A.; Almosbeh, M.J.; AlAbbad, M.T.; AlGhadeer, M.S. Spontaneous umbilical hernia rupture associated with omentum evisceration in a patient with advanced hepatic cirrhosis and refractory ascites. Cureus 2021, 13, e16042. https://doi.org/10.7759/cureus.16042
36. Limbu, Y.; Pokhrel, M.; Bohara, T.P.; Joshi, M.R. Spontaneous rupture of umbilical hernia in an adult with evisceration of small bowel: A case report. J. Soc. Surg. Nepal 2020, 23, 59–62. https://doi.org/10.3126/jssn.v23i2.35842
37. Patel, D.; Dixit, P.; Dave, B.H.; et al. Ruptured umbilical hernia. Indian J. Appl. Res. 2021, 11.
38. Iizuka Y, Hikone M, Shimizu Y, Tanabe M, Sugiyama K, Hamabe Y. Intestinal evisceration and Staphylococcus aureus bacteremia due to ruptured umbilical hernia in a patient with liver cirrhosis: a case report and literature review. Oxf Med Case Reports. 2022 Jul 26;2022(7):omac078. doi: 10.1093/omcr/omac078. PMID: 35903616; PMCID: PMC9318879.
39. Nugroho A, Permata Y, Jamtani I, et al. Emergency presentation of Flood syndrome requiring immediate repair of umbilical hernia: A case report. Int J Abdom Wall Hernia Surg 2022;5:218-20.

40. Tuñón-Féquant, C.; Rodríguez-González, D.; Casanova-Ramos, R.; et al. V-034 Spontaneous evisceration through an umbilical hernia. Br. J. Surg. 2023, 110, znad080.267. https://doi.org/10.1093/bjs/znad080.267
41. Grappolini N, Zanchetta M, Inversini D, Ietto G. Spontaneous bowel evisceration through umbilical hernia in an adult non-cirrhotic patient. BMJ Case Rep. 2024 Apr 8;17(4):e258602. doi: 10.1136/bcr-2023-258602. PMID: 38589238; PMCID: PMC11015293.
42. Jayaprakash A, Kawarat VC, S V. Understanding the Spectrum of Flood Syndrome: A Case Series. Cureus. 2024 Jun 10;16(6):e62059. doi: 10.7759/cureus.62059. PMID: 38989335; PMCID: PMC11234922.
43. Aldemyati, R.; Malaibari, Z. Spontaneous rupture of umbilical hernia without ascites: A case report and literature review. Research Square 2024. https://doi.org/10.21203/rs.3.rs-5194251
44. Barden M, Marinelli D, Cable K. Spontaneous Evisceration, or "Burst Abdomen," in Patient with Prior Flood Syndrome Surgical Repair. Clin Pract Cases Emerg Med. 2025 Jan;9(1):117-119. doi: 10.5811/cpcem.31062. PMID: 39903616; PMCID: PMC12509953.
45. Nicole R Bruton, Ludwig R Fechter and Louis Chadinha. Spontaneous evisceration through an umbilical hernia in an adult. WJCM. 2025. Vol. 7(3):149-150. DOI: 10.18772/26180197.2025.v7n3a9
46. Singh, K.; Lokavarapu, M.; Kumar, N.S.; Singh, A.; Chalise, S.; Kumar, S.; Kumar, A. Management and outcomes of umbilical hernia in decompensated chronic liver disease: A single-unit experience of six cases. Cureus 2025, 17, e82578. https://doi.org/10.7759/cureus.82578
47. Dubovský M, Panyko A, Vidiščák M. Umbilical hernia rupture with small bowel evisceration in advanced cirrhosis (flood syndrome) Surgery Open Science, 2026; 32, 17-18
